# How to Feed the Mammalian Gut Microbiota: Bacterial and Metabolic Modulation by Dietary Fibers

**DOI:** 10.3389/fmicb.2017.01749

**Published:** 2017-09-12

**Authors:** Chiara Ferrario, Rosario Statello, Luca Carnevali, Leonardo Mancabelli, Christian Milani, Marta Mangifesta, Sabrina Duranti, Gabriele A. Lugli, Beatriz Jimenez, Samantha Lodge, Alice Viappiani, Giulia Alessandri, Margerita Dall’Asta, Daniele Del Rio, Andrea Sgoifo, Douwe van Sinderen, Marco Ventura, Francesca Turroni

**Affiliations:** ^1^Laboratory of Probiogenomics, Department of Chemistry, Life Sciences and Environmental Sustainability, University of Parma Parma, Italy; ^2^Stress Physiology Laboratory, Department of Chemistry, Life Sciences and Environmental Sustainability, University of Parma Parma, Italy; ^3^GenProbio s.r.l., Parma, Italy; ^4^Division of Computational and Systems Medicine, Department of Surgery and Cancer, Imperial College London London, United Kingdom; ^5^Department of Food and Drug, University of Parma Parma, Italy; ^6^APC Microbiome Institute and School of Microbiology, National University of Ireland Cork, Ireland

**Keywords:** diet, microbiota, dietary fibers, rat model, acetate, succinate

## Abstract

The composition of the gut microbiota of mammals is greatly influenced by diet. Therefore, evaluation of different food ingredients that may promote changes in the gut microbiota composition is an attractive approach to treat microbiota disturbances. In this study, three dietary fibers, such as inulin (I, 10%), resistant starch (RS, 10%), and citrus pectin (3%), were employed as supplements to normal chow diet of adult male rats for 2 weeks. Fecal microbiota composition and corresponding metabolite profiles were assessed before and after prebiotics supplementation. A general increase in the *Bacteroidetes* phylum was detected with a concurrent reduction in *Firmicutes*, in particular for I and RS experiments, while additional changes in the microbiota composition were evident at lower taxonomic levels for all the three substrates. Such modifications in the microbiota composition were correlated with changes in metabolic profiles of animals, in particular changes in acetate and succinate levels. This study represents a first attempt to modulate selectively the abundance and/or metabolic activity of various members of the gut microbiota by means of dietary fiber.

## Introduction

The term “microbiota” is defined as the community of commensal, symbiotic, and pathogenic microorganisms present in a specific environment (Peterson et al., 2009). Since 2009, different metagenomic projects have shed light on the microbiota composition of humans from various geographical locations (Peterson et al., 2009; Cusack et al., 2013) by assessing hundreds of samples from skin, mouth, and gut. Such projects have generated a massive collection of individual data sets that have provided a detailed view of the composition, diversity, and adaptations of the retrieved bacterial DNA.

Definitely, the most interesting human niche is the gastrointestinal tract (GIT) where microbial colonization occurs at differing efficiency from the oral cavity to the rectum, depending on the different environmental conditions. In the large intestine, an estimated 10^11^–10^12^ bacterial cells/mL (Turroni et al., 2008) gain energy predominantly by fermentation of indigestible dietary components or molecules secreted by the host (Graf et al., 2015).

It is believed that diet modulates the composition of the gut microbiota (Conlon and Bird, 2014; Cockburn and Koropatkin, 2016). In particular, dietary carbohydrates that transit through the GIT are able to influence the composition and stability of the gut microbiota (Fava et al., 2013; Xu and Knight, 2015). Furthermore, disturbing the balance between “protective” versus “harmful” intestinal bacteria may result in a so-called “dysbiosis state” (Tamboli et al., 2004; Milani et al., 2016), where an overgrowth of “pathobionts,” i.e., potentially pathogenic symbionts of the microbiota (Chow et al., 2011), could negatively affect important functions of the microbiota (i.e., maturation and regulation of host immunity and gut functions) (Lee, 2013).

The term “prebiotic” refers to compounds, and in particular to indigestible (i.e., non-digestible by the host) carbohydrates, that are able to stimulate growth and/or metabolism of (selected) beneficial gut bacteria, such as members of the genera *Bifidobacterium* and *Lactobacillus* (Tanaka et al., 1983; Gibson and Roberfroid, 1995; Gibson et al., 2004; Rastall and Gibson, 2015). Such dietary compounds, through their ability to modulate the gut microbiota, are purported to reduce the prevalence and duration of infectious and antibiotic-associated diarrhea and the inflammation and symptoms associated with inflammatory bowel disease, exert protective effects to prevent colon cancer, enhance the bioavailability and uptake of minerals, reduce risk factors for cardiovascular disease, and prevent obesity (Slavin, 2013; Gibson et al., 2017). Thus, the use of prebiotics is considered an important approach to manipulate the gut microbiota in order to prevent or treat unbalanced GIT conditions (Dahiya et al., 2017). Prebiotic compounds, such as the human milk-, galacto-, fructo-, xylo-, and pectin-oligosaccharides and lactulose, are known to selectively stimulate bifidobacterial growth and metabolism (Roberfroid et al., 2010; Cockburn and Koropatkin, 2016; Wilson and Whelan, 2017). However, much less is known about the effects of dietary fibers on the gut microbiota, and it has not been established a direct effect of the supplementation of these compounds on the re-establishment of specific gut commensals. For these reasons, we investigated in this study the effects of various dietary substrates, including inulin (I), resistant starch (RS), and citrus pectin (CP), on the gut microbiota of adult wild-type Groningen rats in a time-limited intervention study employing 16S rRNA microbial profiling and metabolomic analyses.

## Materials and Methods

### Ethics Statement

All experimental procedures and protocols involving animals were approved by the Veterinarian Animal Care and Use Committee of Parma University, and conducted in accordance with the European Community Council Directives dated 22 September 2010 (2010/63/UE).

### Animal Housing

Experiments involved 5/4-month-old male wild-type Groningen rats (*Rattus norvegicus*), originally obtained from the University of Groningen (Netherlands), and bred in animal facilities under standard conditions. This outbred strain displays a large inter-individual variability, as observed in humans (Carnevali et al., 2014). After weaning, rats were housed in same sex sibling groups in rooms under humidity-(50 ± 10%) and temperature-(22 ± 2°C) controlled conditions, a 12-h light–dark cycle (lights on at 7 a.m.), and with food and water available *ad libitum*.

### Diet and Experimental Design

**FIGURE 1:**
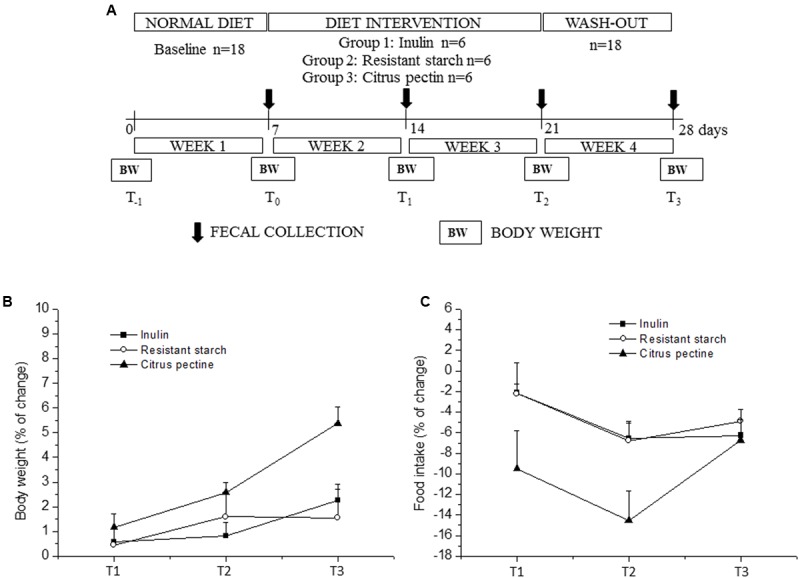
Timeline and results of the experimental procedure. **(A)** shows the schedule of the experimental procedures in wild-type Groningen rats that were fed with different carbohydrate supplements: I (10%), RS (10%), and CP (3%). **(B,C)** Display the body weight and food intake percentage changes relative to the respective “time 0” values, during the experimental procedure, respectively. Values are expressed as means ± SEM. Statistical results are reported in the “Results” section.

**Figure 1A** displays the timeline of all procedures. From the initiation of the experiments, rats were housed individually in polymethyl methacrylate (Plexiglas^®^) cages (39 cm × 23 cm × 15 cm). The first week acts as an acclimatization period, where rats do not change in their habits and continue to follow a normal chow diet. In this way, rats could represent a negative control of themselves, acting as the baseline for microbiota and metabolomics analyses (Turroni et al., 2016). Following this first acclimatization week, rats (*n* = 18) were randomized to standard chow diet with added I (*n* = 6), RS (*n* = 6), or CP (*n* = 6) for 14 days (**Table 1**). Following these 2 weeks, all animals returned to the standard chow diet without added substances, for a 1-week wash-out period before the end of the experiment.

**Table 1:** Fecal samples from rats collected at the different time points.

Samples	Substrates^a^	Concentration (%)	Time points
WT1A – WT6A			T0
WT1B – WT6B	I	10	T1
WT1C – WT6C			T2
WT1D – WT6D			T3
WT7A – WT12A			T0
WT7B – WT12B	RS	10	T1
WT7C – WT12C			T2
WT7D – WT12D			T3
WT13A – WT18A			T0
WT13B – WT18B	CP	3	T1
WT13C – WT18C			T2
WT13D – WT18D			T3

*Dietary fibers supplemented and their concentrations are reported. ^a^: I, inulin; RS, resistant starch; CP, citrus pectin.*

Standard diet consisted of 54.61% nitrogen-free extract (mainly represented by starch and hemicellulose), 5.54% fibers, 19.42% protein, 11.09% water, 2.58% lipids, and 6.76% ash (non-organic mineral matter) (3.9 kcal/g; 4RF21, Mucedola, Italy). The percentage of substrate supplementation to standard chow diet was determined based on published literature: 10% (w/w) I (Van den Abbeele et al., 2011; Pattananandecha et al., 2016), 10% (w/w) RS (Koh et al., 2016), and 3% (w/w) CP (Tian et al., 2016).

Food intake (FI) and body weight (BW) were measured daily and weekly, respectively (**Figure 1A**). BW changes were calculated as the percentage differences between BW values at the end of every week and the respective baseline value (T0). Food consumption was calculated as the percentage changes in FI adjusted for BW (FI/BW) relative to the respective T0 value. The sawdust bedding was replaced at the end of every week and before fecal collection. Consequently, fresh fecal samples were collected manually from a clean sawdust bedding for each animal, at most 1 h after deposition and stored at -20°C until analysis.

### Fecal Bacterial DNA Extraction, 16S rRNA Gene PCR Amplification, and Sequencing

Rats samples were subjected to DNA extraction using the QIAamp DNA Stool Mini Kit following the manufacturer’s instructions (Qiagen). Partial 16S rRNA gene sequences were amplified from extracted DNA using primer pair Probio_Uni and/Probio_Rev, targeting the V3 region of the 16S rRNA gene sequence (Milani et al., 2013). Illumina adapter overhang nucleotide sequences were added to the partial 16S rRNA gene-specific amplicons, which were further, processed employing the 16S Metagenomic Sequencing Library Preparation Protocol (Part #15044223 Rev. B – Illumina). Amplifications were carried out using a Veriti Thermo Cycler (Applied Biosystems). The integrity of the PCR amplicons was analyzed by electrophoresis on a 2200 TapeStation Instrument (Agilent Technologies, United States). DNA products obtained following PCR-mediated amplification of the 16S rRNA gene sequences were purified by a magnetic purification step involving the Agencourt AMPure XP DNA purification beads (Beckman Coulter Genomics GmbH, Bernried, Germany) in order to remove primer dimers. DNA concentration of the amplified sequence library was determined by a fluorometric Qubit quantification system (Life Technologies, United States). Amplicons were diluted to a concentration of 4 nM, and 5 μL quantities of each diluted DNA amplicon sample were mixed to prepare the pooled final Library. Sequencing was performed using an Illumina MiSeq sequencer with MiSeq Reagent Kit v3 chemicals.

### Bioinformatic Analysis

The fastq files were processed using QIIME (Caporaso et al., 2010) as previously described (Milani et al., 2013). Paired-end reads were merged and sequences that had passed quality control were kept with a length between 140 and 400 bp, mean sequence quality score >25, and with truncation of a sequence at the first base if a low quality rolling 10 bp window was found. Sequences with mismatched forward and/or reverse primers were removed.

In order to calculate downstream diversity measures (alpha and beta diversity indices, UniFrac analysis), 16S rRNA Operational Taxonomic Units (OTUs) were defined at 97% sequence homology using uclust (Edgar, 2010) and identified OTUs that were represented by less than 10 sequences were removed. All reads were classified to the lowest possible taxonomic rank using QIIME (Caporaso et al., 2010) and a reference dataset from the SILVA database version 123 (Quast et al., 2013). Biodiversity of the samples (alpha-diversity) was calculated with Chao1 and Shannon indexes. For alpha-diversity analysis, samples with less than 20,000 reads were omitted (see Supplementary Table S1 for details). Similarities between samples (beta-diversity) were calculated by weighted UniFrac (Lozupone and Knight, 2005). Similarity scores were calculated as values between 0 and 1. Principal coordinate analysis (PCoA) representations of beta-diversity were performed using QIIME (Caporaso et al., 2010).

### Metabolomics Analyses

For short-chain fatty acid (SCFA) detection, fecal samples were treated as previously reported (Turroni et al., 2016). Briefly, fecal samples (0.1 g) were dissolved in 800 μL of water and lysed by mechanical treatment with 0.5 g of glass beads (diameter, 106 μm; Carlo Erba, Italy) on a Precellys homogenizer (Bertin, France) at 4°C for 2 min (maximum setting). Supernatant was collected and freeze-dried. Dried fecal extract samples were dissolved in 600 μL of 99.9% D_2_O (Sigma), vortexed for 1 min, and then centrifuged at 18,000 ×*g* for 10 min at 4°C. Supernatant was transferred into a 1.5 mL tube containing 60 μL of 1.5 M potassium phosphate buffer in D_2_O, pH = 7.4, 0.1% 3-(trimethylsilyl)-[2,2,3,3-2H4]propionic acid sodium salt (TSP) and 2 mM sodium azide. The resulting mixture was then vortexed, and 580 μL was transferred to an NMR tube with an outer diameter of 5 mm. ^1^H NMR spectra were acquired using a Bruker 600 MHz spectrometer (Bruker, Rheinstetten, Germany) at the operating ^1^H frequency of 600.13 MHz at a temperature of 300 K. ^1^H one-dimensional NMR profiling was acquired using a presaturation pulse sequence with the form RD-90°-*t*-90°-*t*_m_-90°-ACQ, where RD is the relaxation delay (4 s) during which a low power pulse is applied to saturate the water magnetization, *t* is a short delay typically of about 3 μs, 90° represents a 90° RF pulse, *t*_m_ is the mixing time (10 ms) where a second presaturation sequence and gradients are used to filter out the water signal and ACQ is the data acquisition period (2.7 s). A total of 32 scans were collected into 64 k data points with a spectral width of 20 ppm. Automatic phasing, baseline correction, and reference to TSP signal at (0.00 ppm) were performed in automation using TopSpin 3.6 (Bruker BioSpin, Germany). The processed NMR spectral data were imported into MATLAB (R2016a, 7.14.0.739; MathWorks) and the region containing signals 0.25–10 ppm was digitized into approximately 20 k data points with a resolution of 0.0005 ppm. The remaining water peak region 4.7–4.9 ppm was removed. Probabilistic quotient normalization was applied to the remaining spectral data. Principal component analysis (PCA) and orthogonal partial least squares (OPLS) models were carried out with the unit variance scaling method in SIMCA (P+14.1). OPLS models were built to identify the metabolites with biggest changes in concentration in the fecal water due to the diet intervention. The concentration of these metabolites was calculated when possible due to overlap by integrating the area under the curve of the corresponding signals.

### Statistical Analyses

Two-way ANOVA for repeated measures with “group” as between-subject factor (three levels: I, RS, and CP) was performed for: (i) BW changes, with “time” as within-subject factor (three levels: T1, T2, and T3); (ii) FI-to-BW ratio, with “time” as within-subject factor (four levels: week1, week2, week3, and week4). Follow up analysis was conducted using Student’s “*t*”-test, with a Bonferroni correction for multiple comparisons.

For alpha- and beta-diversity data, PERMANOVA and ANOVA analyses were used, using Tuckey’s HSD *post hoc* test. Linear discriminant analysis (Lefse) was performed for microbial modulation by dietary fibers. Kendall’s tau and significance (two-tailed test) were calculated for co-occurrence analysis. All statistical analyses were performed with SPSS software^1^.

### Data Deposition

The 16S rRNA microbial profiling data sets achieved in this study were deposited in SRA under accession number PRJNA369513.

## Results and Discussion

### Food Consumption and Animal Growth

All dietary supplements examined in this study, i.e., I, RS, and CP, were selected based on previous scientific publications on effectiveness of these compounds in modulating certain gut microbiota members. In fact, I is a well-established prebiotic with stimulating effect on various saccharolytic bacteria (Ramirez-Farias et al., 2009). In addition, RS is the starch fraction that reaches the intestine in an intact form and provides a substantial energy supply for colonic bacteria (Birt et al., 2013). Furthermore, pectin is present in the primary cell walls of dicotyledonous plants, consists of a complex polymer of 1,4-linked-α-D-galacturonic acid units, and can be metabolized by gut bacteria (Martens et al., 2011; Lopez-Siles et al., 2012; Kaya et al., 2014). BW and FI changes are depicted in **Figures 1B,C**, respectively.

Two-way ANOVA for repeated measures yielded a significant effect of time and a time × group interaction for BW changes (time: *p*-value < 0.001; time × group: *p*-value < 0.01) and FI/BW (time: *p*-value < 0.001; time × group: *p*-value < 0.05). BW increment was significantly higher in CP compared to RS group at the end of the experimental protocol (T3). No significant differences among the three groups were found in FI/BW ratio. However, this parameter was significantly reduced in the CP group during the first (FI/BW: 0.0562, *p*-value < 0.01) and second week of diet intervention (FI/BW: 0.0531, *p*-value < 0.001) compared to the relative baseline value (week1, FI/BW: 0.0623).

### Microbial Diversity in Rat Feces after Dietary Fiber Administration

In order to investigate possible gut microbiota modulation exerted by the three different substrates, we assessed the microbiota composition of fecal samples collected from all animals enrolled in this study (a total of 72 fecal samples) based on partial 16S rRNA gene-sequencing analysis as described previously (Milani et al., 2013). The 16S rRNA microbial sequencing of 72 fecal samples produced a total of 4,035,869 sequencing reads with an average of 56,053 reads per sample (Supplementary Table S1). Quality and chimera filtering produced a total of 3,634,488 filtered reads with an average of 50,479 filtered reads per sample (ranging from 1132 to 224,186 reads per sample, Supplementary Table S1).

Assessment of rarefaction curves based on the Shannon and Chao1 biodiversity indexes calculated for 10 subsampling of sequenced read pools indicated that Shannon and Chao1 curves, for all three interventions and at all experimental times, tend to reach a plateau (Supplementary Figures S1a,b). In this case, only 65 samples, those encompassing more than 20,000 reads were considered (see Supplementary Table S1 for details).

Therefore, in all considered cases the obtained sequencing data were deemed adequate to cover the large majority of biodiversity contained within the samples. Moreover, for RS and I experiments, rarefaction curves at time point 0 (aggregate T0, Supplementary Figures S1a,b) were shown to exhibit a significantly (*p*-value < 0.05) higher level of complexity than that observed after fiber interventions (aggregate T1 and T2, Supplementary Figures S1a,b). After the wash-out period, observed differences were shown to be not statistically significant (*p*-value > 0.05). When the analysis was restricted to phylum level, no significant differences were detected for CP intervention (*p*-value > 0.05), comparing rat microbiota before and at the end of the treatment (respectively, aggregate T0 and T2, Supplementary Figures S1a,b).

### Microbiota Modulation by Dietary Fiber Supplementation

**FIGURE 2:**
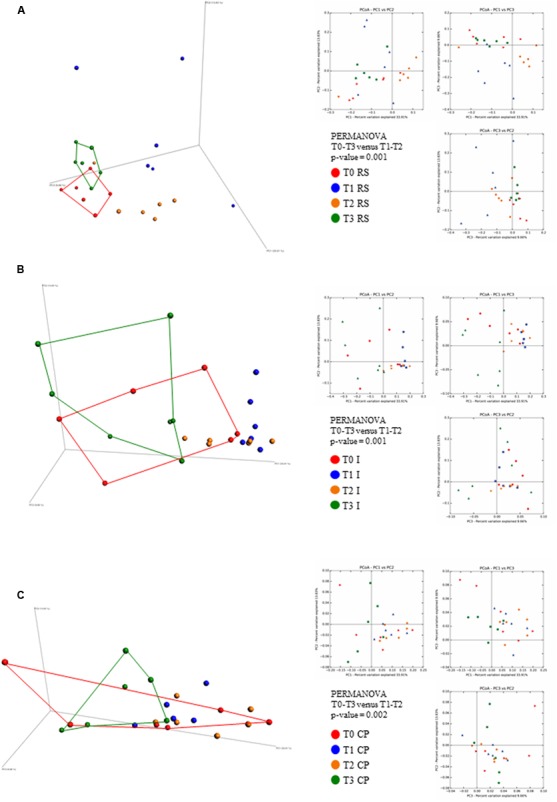
Evaluation of beta-diversity of the three sample sets. The predicted PCoA plots represent weighted UniFrac beta-diversity measures, encompassing all 72 fecal samples, are reported via three-dimensional images as well as two-dimensional sections. The three panels report the compositional analyses obtained for the three dietary fibers supplements (RS, **A**; I, **B**; and CP, **C**) at four different time points and are colored in red for time point 0 (T0), in blue for time point 1 (T1), in yellow for time point 2 (T2), and in green for time point 3 (T3).

In order to evaluate if and how different substrates influence the microbiota composition, we explored the beta-diversity based on weighted UniFrac for RS, I, and CP interventions using the data obtained from samples at the four different time points, after which the UniFrac distance matrix was represented through PCoA (**Figure 2**). Notably, the inter-individual variability of the fecal microbiota assessed by PCoA analysis at T0 revealed a significant uniformity between the various animals enrolled in the study (Supplementary Figure S2a). Moreover, inspection of the predicted taxonomic profiles at phylum level for all samples showed that *Bacteroidetes* (53.9%) represents the dominant phylum, outnumbering the *Firmicutes* (39.8%) and *Proteobacteria* (4%) phyla (Supplementary Figure S2b). *Actinobacteria*, unclassified members (U.m.) of *Saccharibacteria* phylum, *Cyanobacteria* and *Tenericutes* together represent about 2% of the microbiota, while *Verrucomicrobia*, *Spirochaetae*, *Fusobacteria*, and *Elusimicrobia* phyla were determined to exhibit a low level presence (≤0.1%).

Interestingly, for all three substrates, after 1 week of intervention, the samples grouped separately, thus suggesting different effects of these dietary ingredients on the various members of the fecal microbiota (**Figure 2**). The second week of dietary fiber supplementation maintained this trend, with a mitigation of most increased/reduced taxa for I and RS, while a consolidation of taxa that were increased/reduced during the first week of intervention was detected for CP (**Figures 2A–C**).

The wash-out period, consisted of 7 days during which rats were given an unsupplemented chow diet (Warden and Fisler, 2008). Analysis of the corresponding fecal samples indicated a reversion back to the T0 (i.e., baseline) fecal microbial composition (**Figures 2A–C**), thereby abolishing the effects of the administered prebiotics.

Such findings were confirmed by the obtained *p*-value of PERMANOVA statistical analysis (being 0.001 for RS and I and 0.002 for CP experiments), when the two groups data sets (T0 and T3 versus T1 and T2) are compared and excluding samples with less than 20,000 reads (Supplementary Table S1).

### Inulin Selected *Bacteroidetes* – Responsive Taxa

**FIGURE 3:**
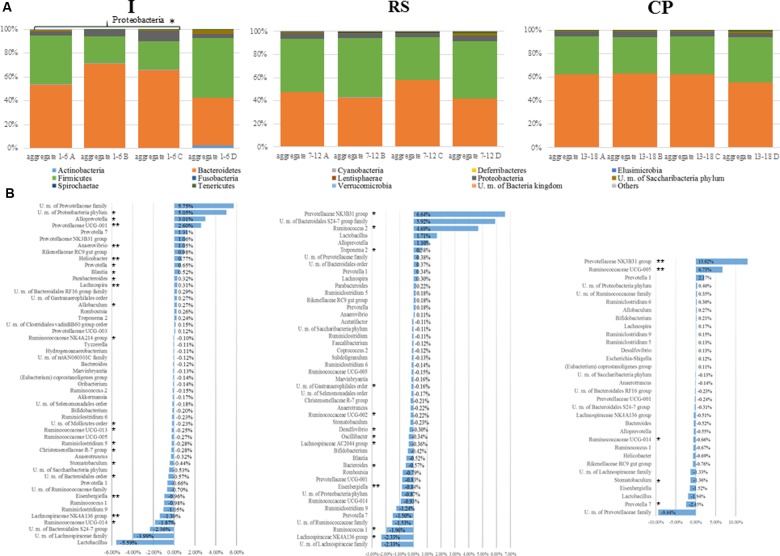
Exploration of microbiota diversity associated with RS, I, and CP interventions at different time points. **(A)** Represents bar plots of the identified bacterial phyla in the 72 analyzed samples, 24 samples per substrate, six for each time point. The legend reports the average relative abundance of each phyla for each substrate tested. **(B)** Depicts the variation in terms of relative abundance >0.1% and <–0.1% of the bacterial taxa, for the three substrates (respectively, I, RS, and CP) at the end of the prebiotic supplementation (T2) in comparison with the initial microbiota composition (T0). Statistical significance of microbial modulation has been calculated using Lefse (https://huttenhower.sph.harvard.edu/galaxy/) and indicated with asterisks as follow: ^∗^*p*-value < 0.05, ^∗∗^*p*-value < 0.005, and ^∗∗∗^*p*-value < 0.001.

Analyses of the microbiota composition following 2 weeks of I intervention, revealed an enrichment in bacterial genera belonging to the *Bacteroidetes* phylum (**Figure 3A**) (from 53 to 65%, not statistically significant) as well as of the *Proteobacteria* phylum (from 3 to 9%, *p*-value = 0.006), with a concomitant decrease of *Firmicutes* (from 41 to 23%, not statistically significant). A similar gut microbiota modulation by I has been described previously (Van den Abbeele et al., 2013; Reygner et al., 2016).

Assessment of modulation of the *Bacteroidetes* phylum members at finer taxonomic levels (**Figure 3B**) revealed an increase in the saccharolytic (Rajilic-Stojanovic and de Vos, 2014) *Prevotellaceae* family members (+13.5%, *p*-value < 0.05) and in *Parabacteroides* spp. (+0.32%, *p*-value = 0.01), while a modest reduction in members of the *Bacteroidales* order was detected (*p*-value < 0.05). When a similar analysis was performed for detected *Firmicutes* phylum members a reduction in *Lachnospiraceae* family members (*Eisengerghiella* spp. – 1% with *p*-value 0.0039, U.m. *Lachnospiraceae* – 4% with *p*-value > 0.05 and *Stomatobaculum* spp. – 0.04% with *p*-value 0.025), *Clostridiales* order members (*Ruminoclostridium* spp. – 1.5% with *p*-value 0.0373 and members of *Ruminococcaceae* family +4.3% with *p*-values < 0.05), and *Christensenellaceae* R-7 group were observed (*p*-value < 0.05) (**Figure 3B**). Interestingly, in accordance with the current exiting literature, the level of *Allobaculum* spp., belonging to the *Erysipelotrichaceae* family, was increased by I treatment (+0.27%, *p*-value = 0.0104) (Catry et al., 2017).

### Resistant Starch Stimulated *Ruminococcus* 2 Group and *Lactobacillus* spp. Growth

Resistant starch supplementation produced a lower modification in the composition of the fecal microbiota at phylum level, as compared to I intervention. In fact, an increase in *Bacteroidetes* was detected (an approximate 10% increase in relative abundance, *p*-value > 0.05), whose main contributors were *Prevotellaceae* family members (+7.5%, *p*-value < 0.05), *Alloprevotella* spp., and *Parabacteroides* spp. (respectively, +1.16 and +0.22%, *p*-values > 0.05). Such findings confirmed previous studies showing that some *Bacteroides* spp. and *Prevotella* spp. can degrade complex plant polysaccharides including starch (Sakamoto and Ohkuma, 2012).

A less evident decrease in members of *Firmicutes* phylum (-10%, *p*-value > 0.05) was reported in accordance with previous studies (Young et al., 2012). Nevertheless, a reduction of *Lachnospiraceae* family members (-5%, *p*-value < 0.05) and *Clostridiales* members (-5.6%, *p*-value < 0.05) was observed as previously reported (Ordiz et al., 2015). Against this trend, *Lactobacillus* spp. and *Ruminococcus* 2 group were increased by RS supplementation (respectively, +1.71 and +4.7%, *p*-values > 0.05 and 0.0065), probably due to the characteristic amylolytic activity of these genera (Abell et al., 2008; Goldsmith et al., 2017). Notably, *Ruminococcus* spp. has been previously shown to play an important role in RS degradation (Walker et al., 2011; Umu et al., 2015). Here, despite an abundant decrease of 2.2% of *Ruminococcus* 1 and *Ruminococcaceae* UCG_002 (*p*-values 0.0249 and 0.0065, respectively, **Figure 3B**), these observations were confirmed (**Figure 3B**).

### Citrus Pectin Selected Specific Taxa, Without Changing the General Microbiota Composition

Citrus pectin intervention was shown to result in weak or undetectable effects on the microbiota composition at phylum level (*p*-value > 0.05). However, when the analysis was performed at lower taxonomic ranks it was possible to identify significant changes in a small number of taxa. Principal variations in rodent microbiota fed with CP were characterized by an increase in members of the *Prevotellaceae* NK3B31 group (+13%, *p*-value = 0.004) and *Ruminococcaceae* UCG-005 (+6.7%, *p*-value = 0.004). Members of the *Prevotella* genus were previously described as active pectin degraders (Nograsek et al., 2015), producing metabolic end-products such as succinate and acetate. Despite this, different OTUs related to the *Prevotella* genus showed different behaviors (**Figure 3B**). Moreover, a significant decrease was observed for *Stomatobaculum* spp. belonging to the *Lachnospiraceae* family (-1.4%, *p*-value = 0.037).

In contrast to the body of data about I and RS, relatively few information are public available for microbiota modulation exerted by pectin. In this context, it has been reported a decrease in the level of *Bacteroidetes* phylum and an increase in *Firmicutes* phylum (Jiang et al., 2016; Tian et al., 2016). In contrast, our experiments revealed no considerable changes in the gut microbiota composition of the rats following CP treatment.

### Effects of Dietary Fiber on Metabolomics Profiles

Short-chain fatty acids and other metabolites derived from degradation of the fibers supplemented to normal chow diet of rats were investigated through NMR analysis and the orthogonal projections to latent structures for discriminant analysis (OPLS-DA) were used to identify discriminative metabolite profiles from samples pre- and post-prebiotic treatment (respectively, T0 and T2 time points). PCA was used to observe the separation trend between the ^1^H NMR metabolic profiles of the fecal water samples for the three groups of animals before and after the diet intervention.

**FIGURE 4:**
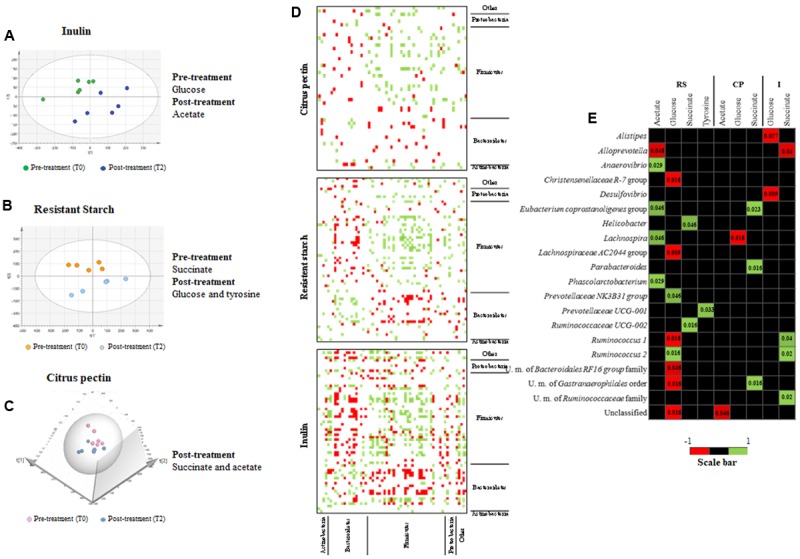
Metabolomics profile of dietary fiber intervention on rats. **(A–C)** Show the PCA score plots of rat fecal samples, respectively, for I (*R*^2^_(cum)_ = 0.57, Q_y(cum)_^2^ = 0.33), RS (*R*^2^_(cum)_ = 0.65, Q_y(cum)_^2^ = 0.414), and CP (*R*^2^_(cum)_ = 0.70, Q_y(cum)_^2^ = 0.408). PCA score plots were obtained considering all metabolites obtained from NMR analysis. The discriminant metabolites between pre- and post-prebiotic intake are reported on the right. Pre-treatment (T0) and post-treatment (T2) samples were considered for the analysis. **(D)** Shows the correlation based on a co-variance analysis depicting the relationship between fecal microbiota members, detected through 16S rRNA profiling analysis. **(E)** Depicts a heat map representation illustrating the co-variance between the bacterial species abundance resulting from 16S rRNA gene-based microbial profiling and the abundance of metabolites resulting from NMR analyses of rat fecal samples. *p*-Values are reported inside each significant square.

Notably, the metabolic profiles show that rats after supplementation with I, RS, and CP clustered separately from the rat gut-metabolomes prior the treatment (**Figures 4A–C**), suggesting an effects of these dietary ingredients on the metabolomes. This finding is likely a reflection of the specific effects on microbiota changes elicited by each of the substrates, and suggests that the modulated bacterial species impact on the associated metabolome (Tang, 2011).

It should be noted that only a relatively small number of metabolites were detected by NMR analysis of the assessed fecal samples, including acetate, succinate, glucose, tyrosine, and *N*-phenylacetylglycine (Supplementary Table S2). However, certain metabolites, such as lactate, butyrate, propionate, valerate, and caproate, which are typically found to be produced by the gut microbiota, appeared to be absent or present at a concentration that was below our detection limit (Sugahara et al., 2015).

The analysis allowed to clearly identify particular metabolites that were characteristic of the pre- or post-treatment groups (**Figures 4A–C**). In this context, a high production of acetate was detected and shown to be discriminative, i.e., able to differentiate two or more sample groups (Weimer and Slupsky, 2013), after supplementation with I and CP (**Figures 4A,C,E**). In contrast, in RS treatment, succinate was the distinctive metabolite present in pre-treatment samples (**Figure 4B**, time point T2). Succinate may be fermented by bacteria enhanced by RS diet, such as those belonging to the *Bacteroidetes* phylum, to produce propionate using the succinate pathway (Reichardt et al., 2014; Rios-Covian et al., 2016).

Moreover, glucose was the second most abundant metabolite and resulted discriminative only in RS post-treatment group (Student’s *t*-test, *p*-value < 0.05) (Supplementary Table S2), probably due to the release of glucose following extracellular RS degradation (Ze et al., 2012). Regarding CP, succinate was the discriminative metabolite of the post-treatment group (**Figure 4C**). Notably, this metabolite may be derived from pectin degradation by *Prevotellaceae* family members (Nograsek et al., 2015).

### Correlations between Microbiota and Metabolite Modulations

In order to assess if a correlation exists between the changes observed in gut microbiota composition following dietary fiber supplementation and the identified metabolite profiles co-variance analyses were performed. Initial OTU-based cluster analysis indicated the existence of co-occurrence/co-exclusion of microbial taxa when a dietary fiber was added to the rodent diet. Therefore, in order to evaluate taxa coexistence, we calculated the Kendall tau rank correlation between the principal genera found in the samples considering only genera showing relative abundance >1% (Turroni et al., 2016; **Figure 4D**). Interestingly, a negative correlation between the genera belonging to the *Prevotella* – *Porphyromonadaceae* group and *Lachnospiraceae* and *Ruminococcaceae* families were detected when rats were fed with the three substrates (*p*-value < 0.05). This association is one of the main detected in the gut microbiota (Faust et al., 2012). In contrast, in our results, *Bacteroides* spp. showed a positive correlation with *Lachnospiraceae* and *Ruminococcaceae* families in RS and I treatments (*p*-value < 0.05).

In order to identify further relationships between metabolite abundances and specific microbe populations, a co-correlation plot was generated, which revealed a variety of significant microbe–metabolite associations (**Figure 4E**). The results highlight a positive covariance between the presence of members of the *Clostridiales* order and acetate production in RS experiments (*p*-values < 0.05, **Figure 4E**). As reported above, RS treatment reduces the presence of genera belonging to the *Clostridiales* family (such as *Anaerovibrio* spp., *Eubacterium* spp., *Lachnospira* spp., and *Phascolarctobacterium* spp.). Consequently, a reduction in this bacterial group may in turn cause a decreased acetate production. Indeed, this metabolite was not represented as discriminative after RS treatment (**Figure 4B**). A positive correlation was detected for *Ruminococcus* 2 and glucose (*p*-value = 0.016), after RS treatment, further confirming the well-known ability of this bacterial genus to degrade starch (Ze et al., 2012). Furthermore, *Prevotellaceae* NK3B31 group showed the identical positive correlation (*p*-value = 0.046). A negative correlation was detected between *Alloprevotella* spp., increased by RS, and acetate (*p*-value = 0.046).

Regarding succinate, a positive correlation was detected for members of the *Ruminococcaceae* family following RS and I supplementations (*p*-values < 0.05, **Figure 4E**). These bacteria were previously described to decrease following supplementation of these fibers (Young et al., 2012), and this finding correlates with the higher presence of succinate in pre-supplementation fecal samples. A negative correlation was detected for *Alloprevotella* spp. and succinate, in I treated rats (*p*-value = 0.04). In CP fecal samples, positive correlations were identified between succinate and *Eubacterium* spp. (*p*-value = 0.023), *Parabacteroides* spp. (*p*-value = 0.016), and U.m. of *Gastranaerophilales* order (*p*-value = 0.016), but these taxa were only modestly modified by this glycan supplementation.

## Conclusion

The disruption of the microbiome equilibrium, which may lead to the so-called dysbiosis state, has been associated with intestinal disorders such as inflammatory bowel disease, irritable bowel syndrome, and celiac disease. There are different strategies proposed to prevent/remedy dysbiosis, one of these being the re-establishment of the microbiota homeostasis through the use of probiotic microorganisms that increase the portion of beneficial bacteria. Unfortunately, a large part of the human gut microbiota is represented so far by unculturable microorganisms, including bacteria that are highly sensitive to oxygen and that are thus very difficult to exploit as probiotic bacteria (Lagier et al., 2016).

An alternative strategy is to selectively increase the number or metabolic activity of specific members of the microbiota by certain food ingredients or prebiotics. In this study, a murine model was fed with food ingredients including non-digestible polysaccharides. Selective and specific modulation of the rat gut microbiota was observed, with enhancement and/or reduction of various gut commensals during treatments with these molecules.

No dysbiosis simulation was performed on the murine model, but the results obtained indicate that dietary fiber, in particular I and RS, could act as valuable prebiotic ingredients for selected microbiota members. The approach described in this study, despite the low number of substrates tested, may represent a valuable procedure in order to assess the microbiota modulation properties of defined food ingredients.

## Author Contributions

CF, RS, AS, and FT designed and performed experiments and wrote the manuscript; LM, GL, and CM performed bioinformatic analyses; LC, SD, MM, AV, BJ, SL, GA, and MD performed experiments; AS, DDR, MV, and FT commented the manuscript; MV, FT, and DvS conceived the study, revised, and approved the manuscript; and all authors reviewed the manuscript.

## Conflict of Interest Statement

The authors declare that the research was conducted in the absence of any commercial or financial relationships that could be construed as a potential conflict of interest.

## Footnotes

1 http://www.ibm.com/software/it/analytics/spss/
