# Supplementary material for: How to Feed the Mammalian Gut Microbiota: Bacterial and Metabolic Modulation by Dietary Fibers
Source: Front Microbiol. 2017 Sep 12;8:1749. doi: 10.3389/fmicb.2017.01749 (PMC5600934; doi:10.3389/fmicb.2017.01749)
Supplement: Supplementary file 1 [file Table_1.PDF]

How to feed the mammalian gut microbiota: bacterial and metabolic modulation by dietary fibers

Chiara Ferrario, Rosario Statello, Luca Carnevali, Leonardo Mancabelli, Christian Milani, Marta Mangifesta, Sabrina Duranti, Gabriele Andrea Lugli, Beatriz Jimenez, Samantha Lodge, Alice Viappiani, Giulia Alessandri, Margerita Dall'Asta, Daniele Del Rio, Andrea Sgoifo, Douwe van Sinderen, Marco Ventura and Francesca Turroni

Supplementary Material

Table S1. 16S rRNA microbial profiling data.

Samples	Number of merged pe reads	Human sequences	Homopolymers > 7	Mismatch in primers >1	Reverse primer not found	Final Read Number
WT01A	169866	0	6	3907	71	165882
WT01B	88854	1	6	1857	164	86826
WT01C	65645	1	1	1556	29	64058
WT01D	88603	32	6	2337	192	86036
WT02A	60749	0	2	1413	21	59313
WT02B	59771	2	4	1482	135	58148
WT02C	72238	1	0	1577	21	70639
WT02D	90705	0	10	1967	179	88549
WT03A	49848	0	1	1018	16	48813
WT03B	85454	2	2	1906	168	83376
WT03C	76087	1	2	1791	17	74276
WT03D	75180	2	8	1420	187	73563
WT04A	58980	0	6	1162	19	57793
WT04B	103654	2	4	2908	245	100495
WT04C	100087	2	5	2300	43	97737
WT04D	92382	4	7	2104	212	90055
WT05A	47609	0	3	1097	17	46492
WT05B	98166	1	6	2603	193	95363
WT05C	101801	2	5	2350	32	99412
WT05D	97967	6	11	1980	231	95739
WT06A	78658	2	4	1733	29	76890
WT06B	229360	10	35	4682	447	224186
WT06C	106684	3	16	2541	64	104060
WT06D	8760	0	3	198	7	8552
WT07A	94847	0	9	2142	38	92658
WT07B	79274	0	4	1692	159	77419
WT07C	107608	2	30	2367	37	105172
WT07D	176932	6	18	3774	433	172701

WT08A	91537	1	4	2028	29	89475
WT08B	61630	1	7	1748	139	59735
WT08C	85174	3	1	1827	16	83327
WT08D	105147	4	17	2873	222	102031
WT09A	107167	6	9	2561	34	104557
WT09B	86942	0	11	1812	215	84904
WT09C	91815	0	6	2188	42	89579
WT09D	78336	2	5	1984	171	76174
WT10A	106431	0	9	2500	36	103886
WT10B	110355	3	8	2562	227	107555
WT10C	76501	0	8	1649	29	74815
WT10D	123615	8	10	2524	262	120811
WT11A	80411	0	12	1736	24	78638
WT11B	96515	0	11	2194	225	94085
WT11C	80316	1	9	1861	33	78412
WT11D	99698	0	9	2054	218	97417
WT12A	60225	0	7	1215	8	58995
WT12B	90459	1	4	2599	189	87666
WT12C	90271	0	7	1937	37	88290
WT12D	104757	0	9	2021	222	102505
WT13A	8342	0	0	190	5	8147
WT13B	81513	2	9	2020	167	79315
WT13C	130217	18	8	2905	128	127158
WT13D	78161	0	9	1854	190	76108
WT14A	12614	1	1	286	14	12312
WT14B	57214	0	4	2303	133	54774
WT14C	125559	4	19	3044	76	122416
WT14D	97157	2	3	3389	178	93584
WT15A	8698	1	1	221	11	8464
WT15B	67252	1	3	1536	84	65628
WT15C	109469	2	5	2504	105	106853
WT15D	73703	4	12	2376	165	71146

WT16A	6288	0	1	139	5	6143
WT16B	65713	2	5	2391	100	63215
WT16C	50941	0	4	1328	32	49577
WT16D	69666	0	5	4071	164	65426
WT17A	42773	2	4	971	31	41765
WT17B	72498	0	6	2262	135	70094
WT17C	1167	0	1	34	0	1132
WT17D	64550	1	12	3377	155	61005
WT18A	27298	2	1	950	28	26317
WT18B	95250	0	6	5436	155	89652
WT18C	3583	0	2	92	2	3487
WT18D	58474	2	2	1502	89	56879

Table S2. Metabolites detected through NMR analysis.

Samples [a]	Acetate (mM)	Glucose (mM)	N-phenylacetylglycine (mM)	Tyrosine (mM)	Succinate (mM)
WT01Apre	4.32	0.97	n.d.[b]	n.d.	0.109
WT01Cpost	5.41	0.92	n.d.	n.d.	0.073
WT02Apre	6.52	1.06	n.d.	n.d.	0.051
WT02Cpost	5.5	1.06	n.d.	n.d.	0.072
WT03Apre	4.07	0.97	n.d.	n.d.	0.097
WT03Cpost	5.11	1.05	n.d.	n.d.	0.078
WT04Apre	5.38	0.74	n.d.	n.d.	0.08
WT04Cpost	5.21	0.85	n.d.	n.d.	0.12
WT05Apre	5.53	1.31	n.d.	n.d.	0.562
WT05Cpost	5.77	0.61	n.d.	n.d.	0.089
WT06Apre	4.3	1.01	n.d.	n.d.	0.15
WT06Cpost	6.56	1.39	n.d.	n.d.	0.085
WT07Apre	4.6	0.67	0.72	0.51	0.11
WT07Cpost	5.53	1.8	0.54	0.39	0.081
WT08Apre	4.83	1.16	0.83	0.67	0.34
WT08Cpost	4.41	2.35	0.57	0.45	0.086
WT09Apre	6.26	0.86	0.66	0.54	0.08
WT09Cpost	5.17	0.98	0.44	0.31	0.074
WT10Apre	4.5	0.97	0.53	0.38	0.092
WT10Cpost	5.01	1.65	0.78	0.58	0.1
WT11Apre	4.18	0.86	0.47	0.35	0.49
WT11Cpost	3.78	1.3	0.39	0.29	0.064
WT12Apre	4.5	0.56	0.4	0.29	0.07
WT12Cpost	5.15	1.77	0.45	0.33	0.074
WT13Apre	5.41	1.1	n.d.	n.d.	0.175
WT13Cpost	5.65	0.99	n.d.	n.d.	0.985
WT14Apre	5.2	1.17	n.d.	n.d.	0.17
WT14Cpost	4.35	0.69	n.d.	n.d.	0.1
WT15Apre	3.94	0.81	n.d.	n.d.	0.127
WT15Cpost	5.65	1.17	n.d.	n.d.	0.09
WT16Apre	4.7	0.72	n.d.	n.d.	0.057
WT16Cpost	6.65	0.4	n.d.	n.d.	0.1
WT17Apre	2.97	0.71	n.d.	n.d.	0.211
WT17Cpost	6.56	1.55	n.d.	n.d.	0.059
WT18Apre	5.59	0.78	n.d.	n.d.	0.082
WT18Cpost	4.84	1.21	n.d.	n.d.	0.07

[a]: in the samples names, “pre” indicates a pre0treatment samples (T0), “post” indicates a post0treatment sample (T2).
[b]: n.d., not detectable.

Supplementary figure legends.

Figure S1. Evaluation of alpha0diversity in I, RS and CP treatment samples. Panel a shows the average rarefaction curve representing variation of the Chao1 diversity index at increasing sequencing depth of the four time points for RS, I and CP samples. Panel b displays the average rarefaction curve representing variation of the Shannon diversity index at increasing sequencing depth of the four time points for RS, I and CP samples.

Figure S2. Basal microbiota composition of wild0type Groningen rats. Panel a reports the beta diversity of fecal microbiota composition of rats at time point 0 (T0), for the three substrates tested. Composition at T0 is reported in red for rats fed with RS, in purple for rats fed with inulin and in light blue for rats fed with CP. Panel b depicts a bar plot showing the average microbiota composition at phylum level of the 18 fecal sample collected at T0 (six samples for each substrate).
