# Supplementary figures and images for: How to Feed the Mammalian Gut Microbiota: Bacterial and Metabolic Modulation by Dietary Fibers
Source: Front Microbiol. 2017 Sep 12;8:1749. doi: 10.3389/fmicb.2017.01749 (PMC5600934; doi:10.3389/fmicb.2017.01749)

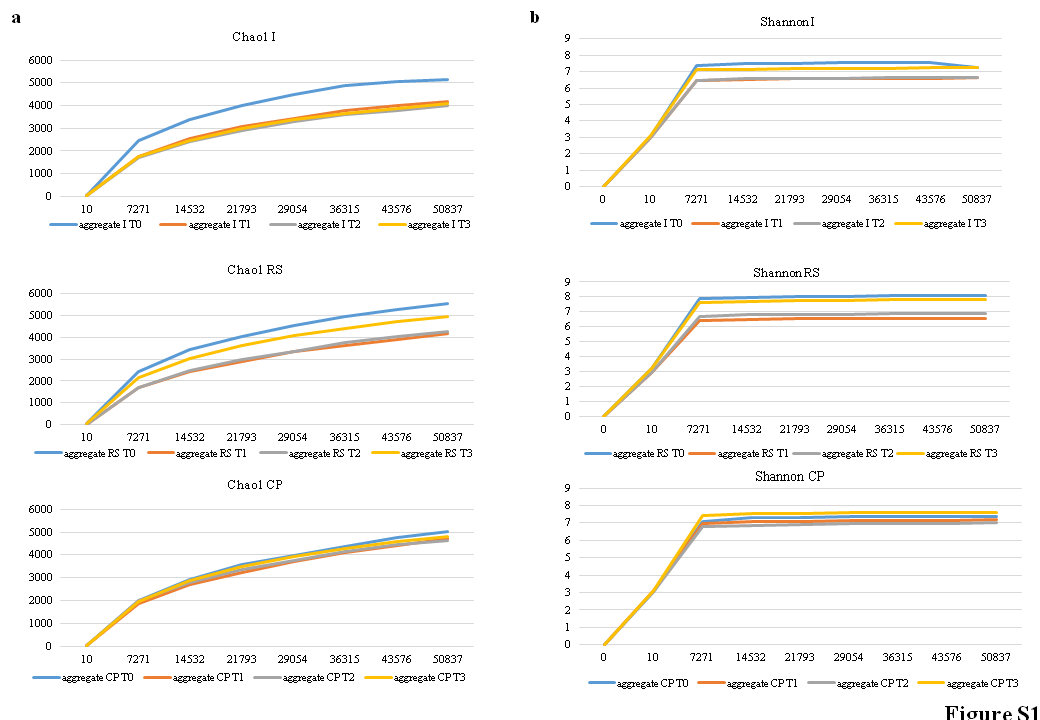

Supplement: Supplementary file 2 [file Image_1.TIF]

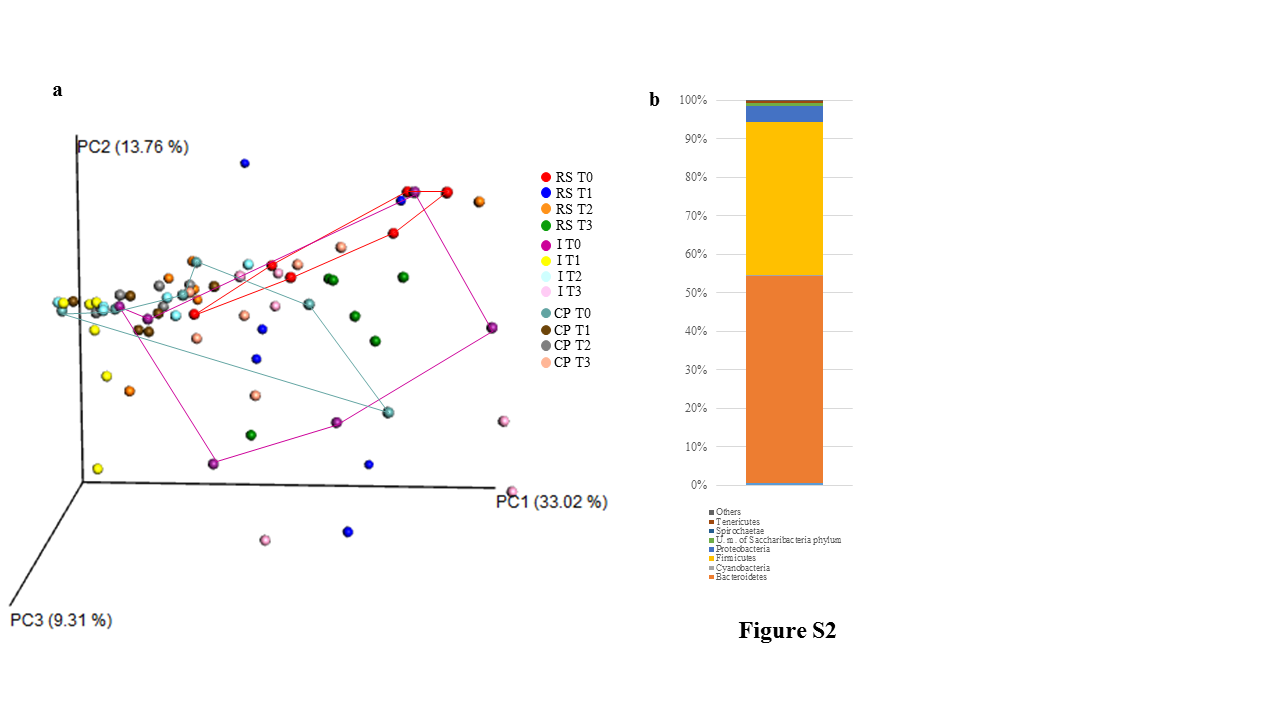

Supplement: Supplementary file 3 [file Image_2.TIF]
